# Maternal transmission of an *Igf2r* domain 11: IGF2 binding mutant allele (*Igf2r*^*I1565A*^) results in partial lethality, overgrowth and intestinal adenoma progression

**DOI:** 10.1038/s41598-019-47827-9

**Published:** 2019-08-06

**Authors:** Jennifer Hughes, Mirvat Surakhy, Sermet Can, Martin Ducker, Nick Davies, Francis Szele, Claudia Bühnemann, Emma Carter, Roman Trikin, Matthew P. Crump, Susana Frago, A. Bassim Hassan

**Affiliations:** 1 Tumour Growth Group, Oxford Molecular Pathology Institute, Sir William Dunn School of Pathology, South Parks Road, OX1 3RE Oxford, United Kingdom; 2 Department of Physiology, Anatomy and Genetics, University of Oxford, South Parks Road, Oxford, OX1 3PT United Kingdom; 3 Department of Organic and Biological Chemistry, School of Chemistry, University of Bristol, Bristol, BS8 1TS UK

**Keywords:** Cancer models, Intrauterine growth, Imprinting

## Abstract

The cation-independent mannose 6-phosphate/insulin-like growth factor-2 receptor (M6P/IGF2R or IGF2R) traffics IGF2 and M6P ligands between pre-lysosomal and extra-cellular compartments. Specific IGF2 and M6P high-affinity binding occurs via domain-11 and domains-3-5-9, respectively. Mammalian maternal *Igf2r* allele expression exceeds the paternal allele due to imprinting (silencing). *Igf2r* null-allele maternal transmission results in placenta and heart over-growth and perinatal lethality (>90%) due to raised extra-cellular IGF2 secondary to impaired ligand clearance. It remains unknown if the phenotype is due to either ligand alone, or to both ligands. Here, we evaluate *Igf2r* specific loss-of-function of the domain-11 IGF2 binding site by replacing isoleucine with alanine in the CD loop (exon 34, I1565A), a mutation also detected in cancers. *Igf2r*^*I1565A*/+*p*^ maternal transmission (heterozygote), resulted in placental and embryonic over-growth with reduced neonatal lethality (<60%), and long-term survival. The perinatal mortality (>80%) observed in homozygotes (*Igf2r*^*I1565A/I1565A*^) suggested that wild-type paternal allele expression attenuates the heterozygote phenotype. To evaluate *Igf2r* tumour suppressor function, we utilised intestinal adenoma models known to be *Igf2* dependent. Bi-allelic *Igf2r* expression suppressed intestinal adenoma (*Apc*^*Min*^). *Igf2r*^*I1565A*/+*p*^ in a conditional model (*Lgr5-Cre*, *Apc*^*loxp/loxp*^) resulted in worse survival and increased adenoma proliferation. Growth, survival and intestinal adenoma appear dependent on IGF2R*-*domain-11 IGF2 binding.

## Introduction

The function of the fifteen homologous extra-cellular domains of the cation-independent mannose 6-phosphate/insulin-like growth factor 2 receptor (M6P/IGF2R or IGF2R) include the binding, trafficking and extra-cellular internalisation of ligands, such as Insulin-like growth factor 2 (IGF2), mannose 6-phosphate (M6P) modified lysosomal proteases and plasminogen^1^. The specific IGF2 and M6P high affinity binding to IGF2R occurs via domain 11, and domains 3, 5 and 9, respectively^1^. The structural basis of the binding interaction of IGF2 and domain 11 of IGF2R has been determined at atomic resolution, including a mechanism to account for structural co-evolution of IGF2 binding to IGF2R in relation to genomic imprinting^2^. With respect to the latter, an exon splicing enhancer (ESE) was identified in monotremes based on ESE dependent changes in the amino acid sequence of the hydrophobic CD loop of IGF2R domain 11. This discovery implied that ESE selection lead to acquisition of IGF2 binding to one of its fifteen domains^2^. Moreover, it also suggested that the evolved and specific IGF2 binding domain of IGF2R was also functionally independent of the other domains of the receptor.

In mammals, *Igf2r* is also a maternally expressed gene (paternal imprinted or silenced), where allele expression is dependent on the ‘parent of origin’^3^. The phenotype of the disrupted (null) maternal derived allele (*Igf2r*^*-m*/+*p*^) in the mouse is associated with over-growth and perinatal lethality, and has been documented by at least three independent groups. The results have all shown placental and embryonic overgrowth by E12.5, a disproportionate overgrowth and tissue specific cardio-pulmonary phenotype and lethality in the early perinatal period, when compared to wild-type littermates^4–10^. The cardio-pulmonary phenotype comprises disproportionate cardiac enlargement relative to other body organs, and pulmonary haemorrhage, with associated generalised oedema. This was proposed as the underlying mechanism of lethality, also observed using a conditional *Igf2r* loxp-null allele^10^. The phenotype also appears strain dependent, but it remains unclear what the functional mechanisms are with respect to associated strain dependent modifier loci^11^. Mammalian *Igf2r* and *Igf2* expression are mainly mono-allelic during post-implantation embryonic and placental tissues, except in humans, where both alleles are frequently expressed (loss of imprinting, bi-allelic expression)^12^. As with the placenta, the embryonic heart is a known site for both *Igf2* and *Igf2r* expression. The implications are that the placenta and heart over-growth are dependent on increased local IGF2 ligand supply. For the heart, the location of *Igf2* expression within the epicardium is proposed to regulate cardio-myocyte growth, supporting a trophic-paracrine mechanism of local IGF2 ligand delivery as opposed to an endocrine effect^13^. The increased Insulin-like growth factor 1 receptor/insulin receptor mediated signaling by increased bioavailable IGF2 arises because of the impaired extra-cellular clearance of IGF2 when IGF2R protein function is disrupted. The increased IGF2 supply following loss of *Igf2r* is also potentially amplified by the combination with increased M6P-lysosomal proteases, especially if the second M6P receptor, the dimeric cation-dependent MPR, is unable to independently rescue M6P-lysosomal supply. The increase in M6P-lysosomal proteases following loss of function of IGF2R can result in release of both IGF2 and IGF1 bioavailable ligands through cleavage of extra-cellular signalling and matrix proteins, such as IGF binding proteins (IGFBP)^14^. Genetic rescue of the *Igf2r*^*−m*/+*p*^ growth and lethality phenotype in the mouse, however, occurs when *Igf2* is co-disrupted, with mice exhibiting the *Igf2* null phenotype of proportionate dwarfism (60% of wild-type), suggesting there is no IGF1 dependent growth rescue^8^.

The relative and specific titration of IGF2 by high affinity binding of M6P/IGF2R domain 11 represents an important growth regulatory mechanism. This evolved mechanism supports the ‘parental conflict’ theory of genomic imprinting. The parental conflict theory proposes that the nutritional resources transmitted to the growing conceptus result from competition between paternal alleles attempting to extract resources for their offspring from the mother (by increasing IGF2 ligand), and from maternal alleles attempting to conserve such resources (by reducing IGF2 ligand through expression of IGF2R)^3,15^. Differential allele expression of *Igf2r* is dependent on methylation of the maternal promoter of an anti-sense long non-coding RNA (lncRNA) termed *Airn*, located within intron 2^16^. Disruption of either the *Airn* promoter or deletion/truncation of *Airn* transcripts in the mouse, results in increased paternal allele *Igf2r* expression relative to the maternal allele, and so bi-allelic expression^9,17–19^. Bi-allelic expression of *Igf2r* following deletion of *Airn* region 2 on the paternal allele, results in a proportional growth reduction in embryos and placenta, again attributed to the sequestration of extra-cellular IGF2 and potentially other ligands^9^. Moreover, the reduced organ specific growth that occurs following IGF2R transgene over-expression has both IGF2 dependent and independent activity^20–22^. The loss-of-function of *Igf2r* that results in disproportionate embryonic overgrowth and lethality in the mouse, is also mirrored following mammalian somatic cell cloning in sheep, where epigenetic suppression of *Igf2r* is thought to contribute to ‘large offspring syndrome’^23^.

The targeting strategy is important to consider when further investigating the phenotypic consequences of the specific loss of function of IGF2R ligand binding domains in the mouse. We previously attempted to generate an allele based on conditional insertion of human *IGF2R* exon 3–48 cDNA, that we could then introduce human mutations into a humanised mouse model. When targeted to the intron 2 region of murine *Igf2r*, this allele resulted in trans-splicing of the knock-in human cDNA with the endogenous maternal mouse *Igf2r* allele, resulting in a hypomorphic allele that exhibited perinatal lethality and disproportionate placental and embryonic growth phenotypes^6^. The additional consequence of this type of allele targeting strategy in the mouse, for both the *Igf2r* and *Igf2* loci, may also be associated disruption of co-expressed miRNAs. For both the *Igf2* and *Igf2r* loci, inadvertent targeting of miRNA has frequently been detected. For example, disruption of the *Igf2* locus can result in loss and gain of both miR-483, and indirectly miR-675 associated with the *H19* non-coding RNA. MiR-675 can negatively regulate IGF1R translation, and so may alter the sensitivity of tissues to IGF2 ligand activation^24,25^. Moreover, *Igf2r* 3′ UTR appears to be a specific target for several miRNAs, such as miR-195 and miR-211^26,27^. Overall, these data suggest more targeted strategies are required in order to directly attribute the entire *Igf2r* loss-of-function phenotype specifically to IGF2 ligand supply alone.

Following our work on the structural and functional basis of the mechanism of IGF2 binding to domain 11 of IGF2R, we have mapped the essential and specific interacting amino acids within the binding domain loops^2^. Here, for the first time, we introduce a direct mouse knock-in mutation of one of the IGF2 binding residues in the binding site CD-loop of domain 11, that results in replacement of isoleucine, a hydrophobic amino acid,with alanine (I15721 in human, I1565 in mouse). This mutation has been detected in human liver cancer^28^, along with other loss-of-function mechanisms such as expansion of a polyG tract in colorectal cancer. It has been proposed that tumour growth promotion might result because of increased local IGF2 supply, as a result of the specific loss of IGF2 binding to IGF2R. The phenotype of this single knock-in allele mutation in the mouse (*Igf2r*^*I1565A*^) resulted in a placental and embryonic over-growth phenotype and partial neonatal lethality, with at least 40% of heterozygous mice surviving into adulthood. As a result of this attenuated phenotype, we were able to further characterise the tumour suppressor function using this novel *Igf2r*^*I1565A*^ allele in adult intestinal adenoma models.

## Results

### Mutation of isoleucine 1565 to alanine results in Igf2r domain 11 specific loss of IGF2 binding

**Figure 1:**
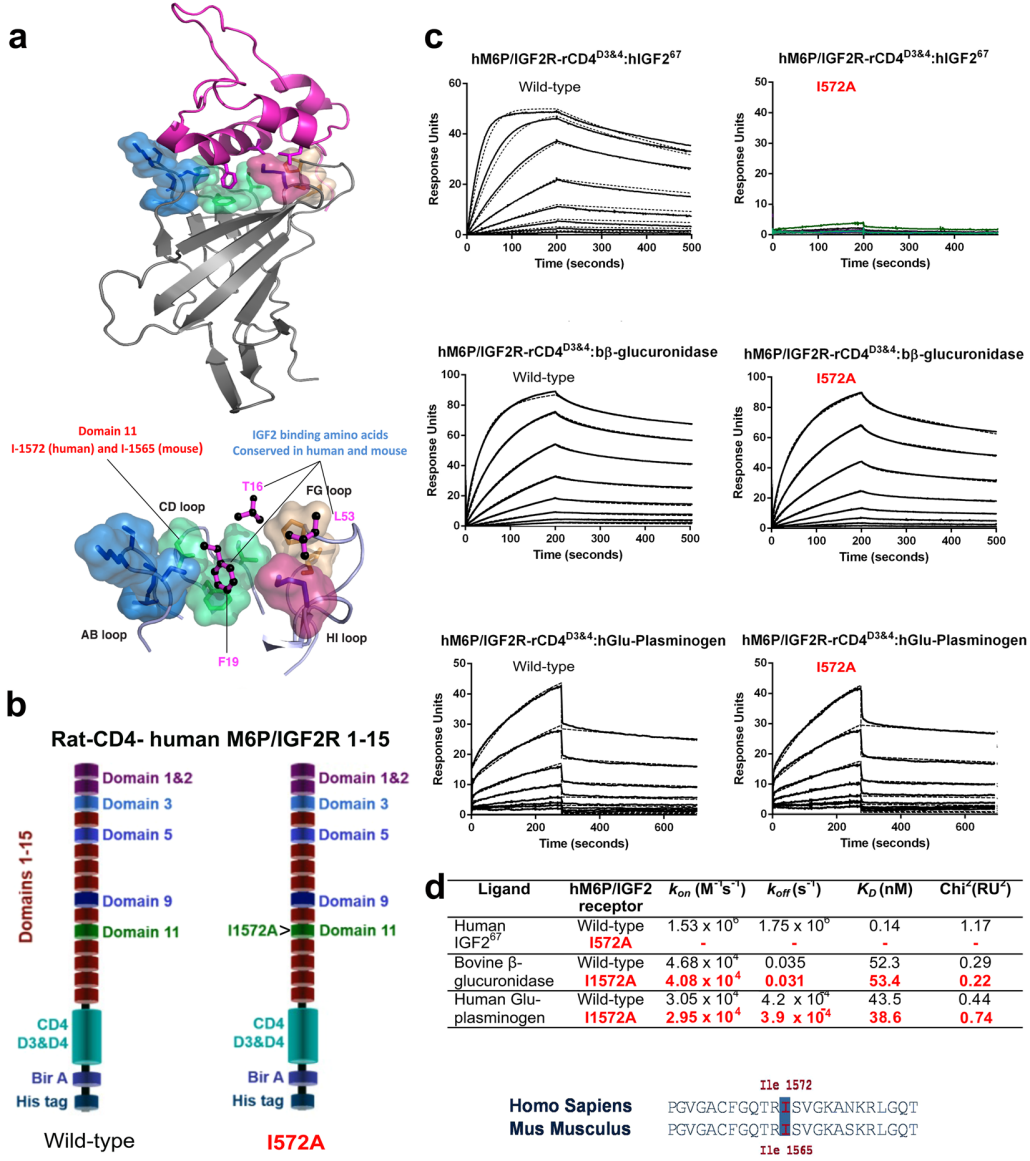
Structural and molecular basis for targeting Isoleucine in the CD loop of domain 11 of M6P/IGF2R. (**a**) Structural configuration of human IGF2 binding to domain 11 of IGF2R (upper panel), including key interacting residues of IGF2 with CD, AB, FG and HI loops of domain 11 (lower panel). (**b**) Generation of a biotin acceptor site, poly-histidine (six histidines) tag and rat CD4 domains 3 + 4 fusion protein with the extra-cellular domains of M6P/IGF2R, with and without (wild-type) mutation I1572A of domain 11. Fusion proteins were expressed, purified and biotinylated as outlined in material and methods and Supplementary Fig. 1. (**c**) Affinity of wild-type and I1572A mutated M6P/IGF2R to human IGF2 using surface plasmon resonance. Sensorgram of the binding interactions with IGF2 (upper panel), beta-glucuronidase for M6P (middle panel) and plasminogen (lower panel), using a 1:1 binding model. Note lack of binding of IGF2 to I1572 mutant. (**d**) Association rates, dissociation rates and affinity constants for wild-type and I1572A interaction with ligands, including Chi^2^ values of their respective fits. Line up of ‘TRIS’ CD loop sequence and designation of I1572 (human) and I1565 (mouse).

Prior to generating a knock in allele of mouse *Igf2r*, we first established that the intended mutation results in loss of receptor IGF2 binding. A high degree of evolutionary conservation of IGF2R domain 11 sequence and structure between human and mouse allowed us to map mutations of the human binding site to those of the mouse^2^. The amino acid residue I1572 (mouse I1565) is a key residue in domain 11 that orientates a conserved CD loop hydrophobic residues with respect to F19 and T16 of IGF2 (Fig. 1a). We introduced the I1572A mutation into human extra-cellular domains (1–15) fused at the C-terminal to rat CD4 (domains 3 + 4), biotin acceptor domain and histidine tag, so that we could purify recombinant mutant protein for affinity studies (Fig. 1b). Following transient co-expression in HEK293T cells with biotin ligase expression vectors, wild-type (WT) and mutated (I1572A), human proteins were purified using affinity to a nickel column, followed by immobilisation on a streptavidin coated BIAcore biosensor chips. Binding kinetics to human IGF2, M6P-beta-glucuronidase and plasminogen were determined using surface plasmon resonance. The I1572A mutation significantly reduced IGF2 binding by 100-fold, yet the 1–15 protein domains bound M6P and plasminogen with similar affinities, indicating that the I1572A mutation was specific for IGF2 binding alone (Fig. 1c,d).

### Maternal transmission of the Igf2r I1565A knock-in allele results in lethality

**Figure 2:**
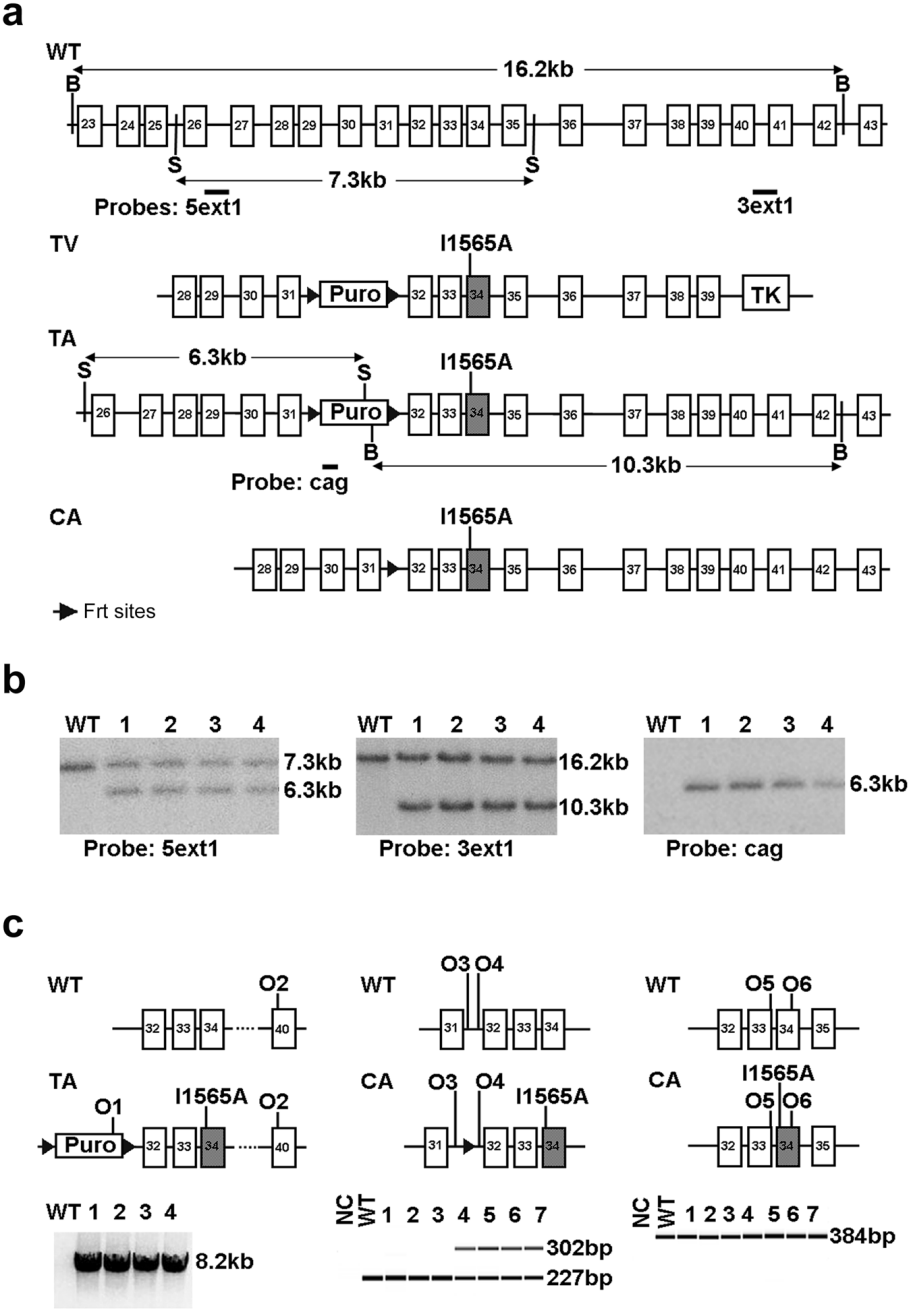
Knock-in strategy of Igf2r I1565A allele, germ-line targeting and genotyping. (**a**) Outline of the *Igf2r* I1565A targeting strategy. Gene exons are shown as boxes (exon 34 shaded), FRT sites as large triangles, WT, wild type; TV, targeting vector; TA, targeted allele; CA, combined allele; Puro, puromycin resistance cassette, TK, thymidine kinase, 5ext1, 3ext1, cag, Southern probes, O1-6, oligos 1–6 (**b**) To detect correct homologous recombination at the 5′ end of the construct, 5 clones of ES cells were digested with PspOMI and hybridised to probe 5ext1 (left panel), 4 clones gave the expected band at 6.3 kb. For the 3′ end of the construct, cells were digested with NsiI and hybridised to probe 3ext1 (central panel), all 4 gave the expected band at 10.3 kb. To detect a single insertion site, clones were digested with NsiI and hybridised to probe cag, and all 4 gave a single band at the correct size of 6.3 kb (right panel). (**c**) Genotyping PCR analysis on pups bred from clone 2 in (**b**) (Left panel); 8.2 kb fragment of TA amplified by oligos 1 and 2 denotes the presence of full complement of exons downstream of I1565A. (Middle panel; following cross with Flp deleter to generate CA); 302 bp fragment amplified by oligos 3 and 4 denotes the presence of the I1565A knock-in allele, and is the basis for genotyping. (Right panel; following cross with Flp deleter to generate CA); 384 bp fragment amplified by oligos 5 and 6 denotes identical product size for both wild-type I1565A knock-in allele.

A mouse *Igf2r* targeting vector with exon 34 mutation I1565A was recombined in C57BL6 ES lines, and transformed under positive (puromycin) and negative selection with ganciclovir (Fig. 2a). Southern blots using 5′ and 3′ probes, confirmed orientation of a single copy insert (Fig. 2b, see Supplementary Figs S1–S3 for full blots). Following PCR verification of correct targeting, two lines were bred with a Cre-deleter strain to remove the puromycin selection cassette, but retaining the flp sites. Two lines achieved germ-line transmission, with line 2 being the basis of subsequent experimental breeding (Fig. 2b,c., see Supplementary Fig. S4 for *Igf2r* PCR).

**Figure 3:**
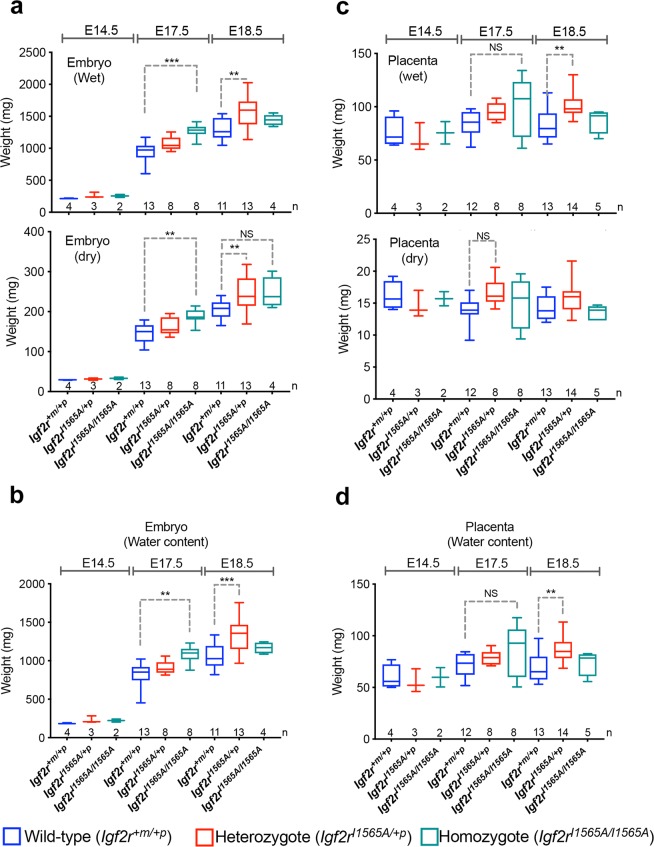
The effects of maternal transmission of *Igf2r*^*I1565A*/+*p*^ on embryonic and placental growth. (**a**) Comparison of heterozygote (*Igf2r*^*I1565A*/+*p*^) and homozygote (*Igf2r*^*I1565A/I1565A*^) embryo wet and dry weights following maternal transmission of *Igf2r*^*I1565A*/+*p*^. PCR confirmed genotypes at E14.5, E17.5 and E18.5. Number of embryos per genotype and time-points are shown along the x-axis. Wet weight; E17.5 ***p < 0.0001, E18.5 **p = 0.0001. Dry weight; E17.5 **p = 0.007, E18.5 **p = 0.0038, NS p = 0.089. (**b**) Water content (WC = wet weight – dry weight) for data in (**a**). E17.5 **p = 0.012, E18.5 ***p = 0.0002. (**c**) Comparison of wet and dry weights of placentas following maternal transmission of *Igf2r*^*I1565A*/+*p*^ resulting in heterozygote and homozygote genotypes as in (**a**). Wet weight; E17.5 NS p = 0.0566, E18.5 **p = 0.0054. Dry weight; E17.5 NS p = 0.0620. (**d**) Water content for data in (**c**). E17.5 NS p = 0.1522, E18.5 **p = 0.0061. Horizontal line = mean, box plots 95% ± whiskers maximum and minimum. For statistical analysis, one-way ANOVA with Bonferroni multiple comparisons test. NS-not significant.

Previously reported germ-line *Igf2r* loss of function alleles identified a placental and embryonic overgrowth phenotype (120–135%) detectable by E12.5^7,8,29^. We first evaluated embryonic growth following timed matings using maternal transmission of the knock-in *Igf2r* allele (*Igf2r*^*I1565A*/+*p*^). Maternal transmission of either the heterozygote *Igf2r*^*I1565A*/+*p*^ or homozygote allele (*Igf2r*^*I1565A/I1565A*^), resulted in normal Mendelian segregation *in utero* based on genotyping of 86 pups (see Supplementary Table 1). Despite litter variation, embryo growth (wet and dry weight) appeared significantly greater in *Igf2r*^*I1565A*/+*p*^ heterozygote (114–122%, p < 0.0001) and *Igf2r*^*I1565A/I1565A*^ homozygote (118–135%, p = 0.0001) mice compared to that in wild-type littermates (Fig. 3a). An increase in water content was also observed by E17.5, and remained high in heterozygotes by E18.5 (p = 0.0002, Fig. 3b). For placental growth, there appeared greater heterogeneity between genotypes compared to embryos (Fig. 3c), but with an overall increase in water content observed at E18.5 in heterozygotes (112–118%, p = 0.0061, Fig. 3d). Overall, in comparison of the incremental growth with respect to genotype observed in embryos, placenta growth appeared more variable in respect of significant gain in dry weight and water content (Fig. 3).

**Figure 4:**
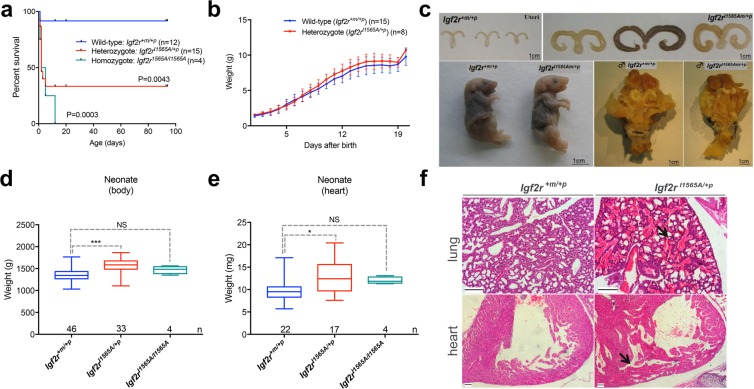
The effects of maternal transmission of *Igf2r*^*I1565A*/+*p*^ on survival and postnatal growth growth. (**a**) Kaplan-Meier survival plots following maternal transmission of *Igf2r*^*I1565A*/+*p*^ resulting in heterozygote and homozygote PCR confirmed genotypes. Comparison of wild-type and heterozygote *Igf2r*^*I1565A*/+*p*^, (p = 0.0043), and comparison of wild-type and homozygote *Igf2r*^*I1565A/I1565A*^, (p = 0.0003 Log-rank, Mantel-Cox test). (**b**) Postnatal weights of surviving littermates (±S.D.) from birth to 21 days. Comparison of wild-type and heterozygote *Igf2r*^*I1565A*/+*p*^ growth was non-significantly different (p = 0.68). (**c**) Gross appearances of neonatal and adult (6–8 weeks) wild-type and heterozygote *Igf2r*^*I1565A*/+*p*^ littermates; female uteri (upper panel), new born pups (lower left panel) and male genitalia (lower right panel). (**d**,**e**) Comparison of neonatal body weight (**d**) and heart weight (**e**) for wild-type, *Igf2r*^*I1565A*/+*p*^ and *Igf2r*^*I1565A/I1565A*^ genotypes. Note that the surviving homozygote *Igf2r*^*I1565A/I1565A*^ were not significantly different from wild-type (NS-body weight p = 0.2470, NS-heart weight p = 0.1583), whereas the heterozygotes *Igf2r*^*I1565A*/+*p*^ genotyped pups were significantly heavier (***body weight p < 0.0001, *heart weight p = 0.008). Horizontal line = mean, box plots 95% ± whiskers maximum and minimum. Unpaired two-way t-test. (**f**) H & E stained formalin fixed and paraffin embedded sections through lungs (upper panel) and hearts (lower panel) for wild-type and *Igf2r*^*I1565A*/+*p*^ pups that died at birth. Note the alveolar and bronchiole haemorrhage (arrow, upper panel) and the thickened and trabeculated ventricular wall muscle (arrow, lower panel) in *Igf2r*^*I1565A*/+*p*^.

Following evaluation of 20 litters and 182 pups at birth (perinatal), maternal transmission of the *Igf2r*^*I1565A*/+*p*^ heterozygote allele resulted in normal Mendelian segregation (Table 1). Following breeding to generate homozygotes, however, perinatal homozygote *Igf2r*^*I1565A/I1565A*^ pups were significantly depleted in number (~20% of expected pups, Table 1, p = 0.0006, *X*^2^ = 14.97). Subsequent survival of older pups (neonates) revealed significantly decreased survival of heterozygotes *Igf2r*^*I1565A*/+*p*^ (40%) following maternal transmission (log-rank test, p = 0.0043), and significantly worse survival for *Igf2r*^*I1565A/I1565A*^ homozygotes (log-rank test, p = 0.003), with all the latter pups failing to reach weaning (Fig. 4a). Subsequently, whole body growth (weight) of the surviving *Igf2r*^*I1565A*/+*p*^ heterozygote pups appeared slightly higher than wild-type littermate controls, with a similar trajectory of growth with time, yet this was not statistically different (p = 0.68, Fig. 4b). We interpret these observations with respect to variability in the over-growth phenotype, with pups with the greater over-growth less likely to survive into adulthood. Overall, neonatal whole body weights were greater in surviving heterozygotes *Igf2r*^*I1565A*/+*p*^ compared to wild-type littermates (p < 0.0001, Fig. 4d).

**Table 1:** Summary of breeding outcomes at birth.

*Igf2r*^*I1565A*^ allele transmission	Cross	Genotypes	Observed	Expected	
Maternal	*Igf2r*^+*m/I565A*^ ♀ × *Igf2r*^+*m*/+*p*^ ♂	*Igf2r* ^+m/+p^	39	34.5	*P = NS
	(11 litters, 5 pairs)	*Igf2r* ^*I565A*/+ *p*^	30	34.5	
Paternal	*Igf2r*^+*m*/+*p*^ ♀ × *Igf2r*^+*m/I565A*^ ♂	*Igf2r* ^+m/+p^	25	27	*P = NS
	(10 litters, 3 pairs)	*Igf2r* ^+ *m/I565A*^	29	27	
Homozygote	*Igf2r*^+*m/I565A*^ ♀ × *Igf2r*^+*m/I565A*^ ♂	*Igf2r* ^+m/+p^	25	14.75	*P = 0.0006 *X*^2^ = 14.97
		*Igf2r* ^+ *m/I565A*^ *or Igf2r* ^*I565A*/+ *p*^	30	29.5	
	(9 litters, 3 pairs)	*Igf2r* ^*I565A/I565A*^	4	14.75	

^*^X^2^ and one-way ANOVA, Kruskal-Wallis, with Dunns multiple comparison post-test.

### Maternal transmission of the Igf2r I1565A knock-in allele results in tissue specific growth

Heterozygote *Igf2r*^*I1565A*/+*p*^ exhibited evidence of increased organ growth (Fig. 4). Uteri of surviving female heterozygote adult mice appeared grossly enlarged and fluid filled compared to wild-type littermates, consistent with a degree of vaginal atresia, whereas male genitalia appeared proportionate (Fig. 4c). We did not observe extra post-axial digits or a kink in the tail of heterozygote mice, phenotypes previously associated with null alleles of *Igf2r*. Neonatal heart weights were also greater in surviving heterozygotes *Igf2r*^*I1565A*/+*p*^ (p = 0.008, Fig. 4e). Even though there was a subjective appearance of being larger, dehydration and cadaver deterioration meant we could not accurately determine whole body weights in non-surviving (heterozygote and homozygote) pups at the time of death. The relative proportion of the heart to body weights were non-significantly different in both surviving heterozygotes (0.79%, p = 0.69) and homozygotes (0.81%, p = 0.62), compared to wild-type controls (0.76%), supporting some degree of proportionality of heart growth (paired t- test of log_10_-transformed ratios). Histological examination revealed enlarged hearts with expanded trabecular areas, with extensive regions of haemorrhage in the lungs of heterozygotes *Igf2r*^*I1565A*/+*p*^ compared to wild-type controls. Overall, the heart-lung phenotypes we assumed to account for perinatal and neonatal mortality in the larger compared to the smaller pups (Fig. 4f). As the assumption was that the heart growth phenotype was due to increased IGF2 supply, we next considered an approach to reverse the heart phenotype, and potentially survival, by using a conditional *Igf2* allele combined with a heart specific *Cre* knock-in line (*Nkx2,5.Cre*). By conditional generation of loss of *Igf2* expression in the heart, we would expect the lethality of *Igf2r*^*I1565A*/+*p*^ to be rescued. Published data has previously shown that the origin of heart *Igf2* expression to be mainly from the epicardium, and that by combining *Nkx2,5.Cre* and *Igf2*^*loxp/loxp*^, ventricular wall thickness (as a measure of cardiac growth) can be specifically reduced^13,30^. Following breeding with *Igf2r*^*I1565A*/+*p*^ and generating 4 litters and 33 pups, we failed to identify progeny with combined *Igf2r*^*I1565A*/+*p*^ and *Nkx2,5.Cre* genotypes (not shown). We attributed this outcome to a low statistical likelihood of allele segregation, as both *Nkx2.5* and *Igf2r* are both co-located on mouse chromosome 17 (Chr17:26838664, Chr17:1268406, respectively).

**Figure 5:**
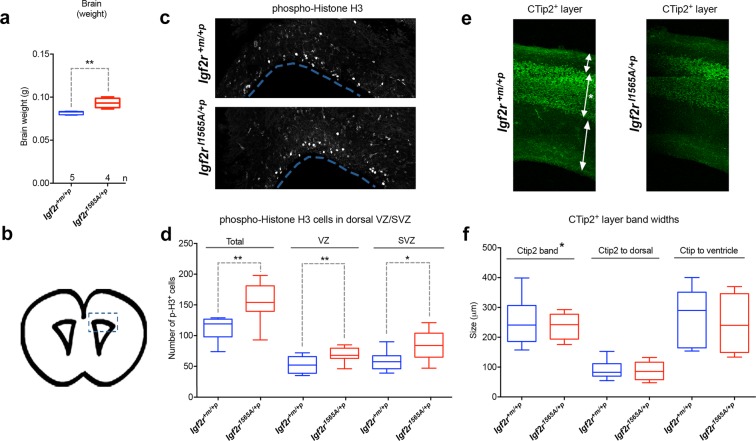
The effects of maternal transmission of *Igf2r*^*I1565A*/+*p*^ on brain growth. (**a**) Brain weights at E18.5 are significantly increased in heterozygote *Igf2r*^*I1565A*/+*p*^ compared to wild-type littermates (**p = 0.0079 Mann-Whitney test). (**b**) E18.5 mouse developing brain coronal image derived from the Allen Developing Mouse Brain Atlas (http://developingmouse.brain-map.org/experiment/siv?id=100054035&imageId=101103438&initImage=nissl). Box indicates the cerebral cortex region of the lateral ventricle in which the pH3 positive cell numbers in (**c**) were assessed. (**c**) Immuno-localisation in 50 µm coronal brain sections of phospho-histone H3 at E18.5 in wild-type and *Igf2r*^*I1565A*/+*p*^ heterozygous littermates. (**d**) Quantification of pH3 positive cell numbers obtained from confocal images (z-stacks) of the ventricular zone (VZ), subventricular zone (SVZ) and in both of these regions (Total) from single brain sections of wild-type (n = 8) and *Igf2r*^*I1565A*/+*p*^ (n = 9) littermates. Proliferation as judged by this biomarker was increased in both the VZ and SVZ in heterozygote *Igf2r*^*I1565A*/+*p*^ compared to wild-type littermates (**Total p = 0.0025, **VZ p = 0.0084, *SVZ p = 0.0117, unpaired one-tailed t-test). (**e**) Immuno-localisation in 50 µm coronal brain sections of cortical layer marker, CTip2, at E18.5 in wild-type and *Igf2r*^*I1565A*/+*p*^ heterozygous littermates. (**g**) Quantification of the relative size and position of the CTip2 positive band in wild-type (n = 8) and *Igf2r*^*I1565A*/+*p*^ heterozygous (n = 9) littermates. (Non-significant; Ctip2 p = 3365, Ctip2 to dorsal p = 0.4040, Ctip2 to ventricle p = 0.5989, unpaired one-tailed t-test). Scale bars 100 µm. Box plots: horizontal line = mean, box 95% ± whiskers maximum and minimum.

Brain weight was also proportionately greater (at E18.5) in heterozygotes *Igf2r*^*I1565A*/+*p*^ compared to wild-type controls (p = 0.0079, Fig. 5a). Quantification of phospho-histone H3 (pH3) in ventricular coronal sections revealed significant increased labelling in the sub-ventricular and ventricular zones of the embryonic cerebral cortex, consistent with stimulation of cell proliferation of radial glial stem cells and intermediate progenitors, respectively, in heterozygote *Igf2r*^*I1565A*/+*p*^ (Fig. 5b–d). We examined cortical layering with the layer V/V1 marker, CTip2, and did not observe changes in position or thickness of CTip2+ cells, suggesting that migration was unaltered (Fig. 5e,f).

Maternal transmission of the knock-in allele *Igf2r*^*I1565A*/+*p*^ resulted in a mainly proportionate over-growth phenotype and partial neonatal lethality. Since it was only the IGF2 binding capacity of the IGF2R that was disrupted, these phenotypes can be attributed to increased supply of IGF2 ligand. We next attempted to quantify the total amount of tissue IGF2 using Western blots from tissue extracts (placenta, embryo and heart) using an anti- mouse IGF2 antibody. Following electro-transfer, we noted that the pH of the buffer altered whether the mature (7.5 KDa) isoform of IGF2 was transferred compared to the immature (Big-IGF2) isoforms that were also detectable^31^. Despite these differences, we could not reliably determine any increase in IGF2 in the *Igf2r*^*I1565A*/+*p*^ tissues compared to littermate wild-type controls (data not shown). As the processing of IGF2 away from the cell signaling receptors at the tissue level (IR and IGF1R) accounts for the function of IGF2/M6P receptor and the mouse phenotypes, the total level of IGF2 in the tissue may not be representative of the metabolism of IGF2 in the extra-cellular space, such as following the sequestration of IGF2 by IGF binding proteins. As our phenotype is more subtle and partially lethal compared to the lethality in the complete *Igf2r* knock-out mouse, we suspect that we do not have the analytical assays of sufficient sensitivity to identify what maybe a less than two-fold effect on IGF2 levels between genotypes. The *Igf2* gene is expressed *in utero*, and not in adult mice, and this might also account for the growth and prolonged survival of a proportion of heterozygote pups, as the wild-type paternal *Igf2r* allele may be expressed at lower relative levels but be sufficient for compensation of the loss of function on the maternal allele. The timing of IGF2 bio-availability may therefore complicate the analysis of when to measure IGF2 levels, as our previous evidence suggests the majority of mouse embryonic growth *Igf2* dependent effects occur between E9-E10 of mouse gestation^32^. Timing of measurement is supported by the homozygote *Igf2r*^*I1565A/I1565A*^ pups that do not express a wild-type *Igf2r* paternal allele, but exhibited the more severe late gestation phenotype and early perinatal mortality.

### Paradoxical effects of Igf2r allelic dosage in the ApcMin intestinal adenoma model

**Figure 6:**
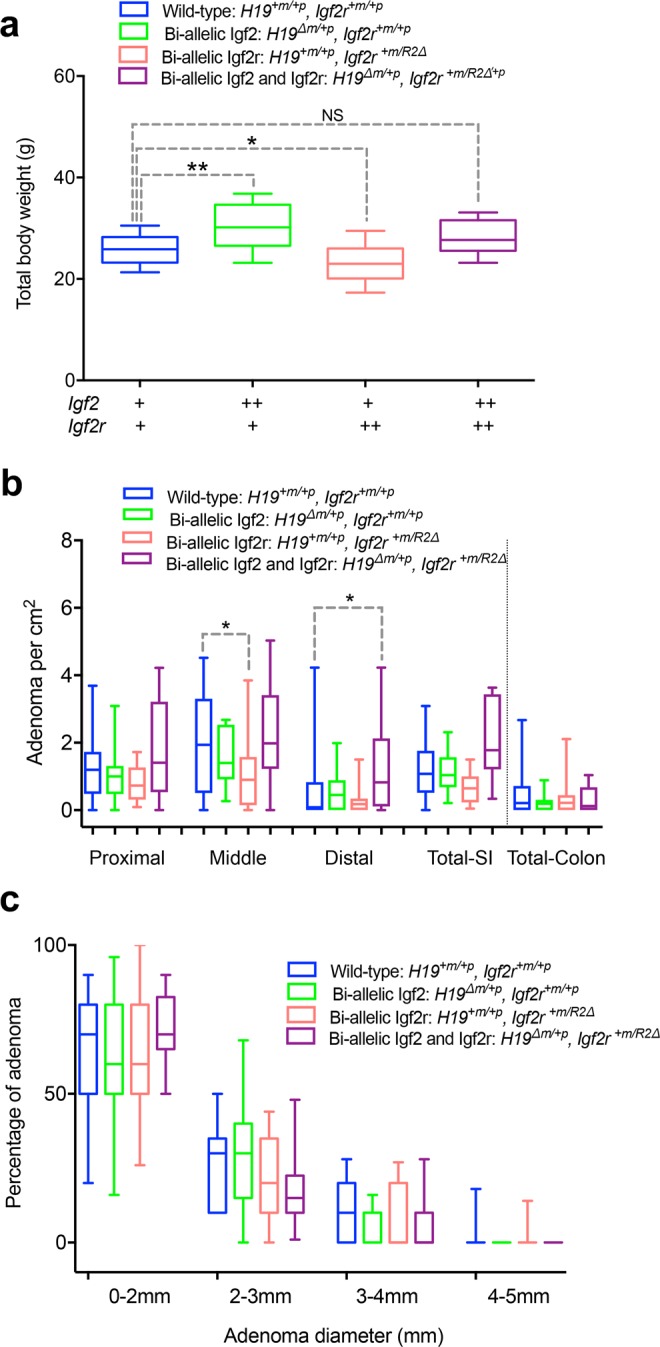
Gain of function of *Igf2r* (bi-allelic expression) suppresses intestinal adenoma number in *Apc*^*Min*/+^. (**a**) Adult body weights (6–8 weeks) of littermates (on an *Apc*^*Min*/+^ background) derived from a cross combining bi-allelic expression of *Igf2* via a 13Kb deletion of the imprinting control region (*H19*^*Δm*/+*p*^ or ++), resulting in over-growth (p = 0.002), with bi-allelic expression of *Igf2r*, via disruption of *Airn* imprinting control region (*Igf2r*^+*m/R2Δ*^ or ++) resulting in decreased growth (p = ,0.0326). Combined genotypes resulted in rescue of *Igf2* mediated overgrowth by bi-allelic (gain of function) of *Igf2r*, such that littermates were non-significantly different from wild-type (+) controls (p = 0.2174). One-way ANOVA with Bonferroni multiple comparisons test. (**b**) Comparison of intestinal adenoma number controlled for body growth (per cm^2^ of intestine) at postnatal day 120 in the proximal, middle and distal thirds of the small intestine (SI) and colon between genotypes in (**a**). Note the wide variation precluding significant differences in the means. (*Middle p = 0.0187, *Distal p = 0.0183, One-way ANOVA with Bonferroni multiple comparisons test following removal of 10 outliers using ROUT (Q = 1%) and confirmation of normality (Kolmogorov-Smirnov test). Overall suppression of adenoma numbers in *Igf2r*^+*m/R2Δ*^ and the paradoxical significant increase in adenoma only when combined with *H19*^*Δm*/+*p*^. (**c**) Comparison of adenoma diameter with the associated genotypes in (**a**,**b**). Note non-significant differences in adenoma size suggesting genotypic effects may be acting during early phase of adenoma development (p > 0.999 for all comparisons). Box plots: horizontal line = mean, box 95% ± whiskers maximum and minimum.

Mutation and loss of heterozygosity of *IGF2R* occurs in human cancers, including the mutation I1572A in domain 11^28,33–36^. In order to further evaluate the potential tumour suppressor function of *Igf2r* dependent IGF2 regulation, we utilised *Igf2r*^*I1565A*/+*p*^ in a human early stage cancer mouse model. We first validated an intestinal adenoma model based on *Apc*^*Min*/+^ murine model of Familial Adenomatous Polyposis. The *Apc* Min point mutation results in strain dependent development of beta-catenin driven multiple intestinal adenoma that can be easily quantified by counting from the proximal, middle and distal small intestine, to caecum and colon, all at post-mortem. When *Apc*^*Min*/+^ was combined with bi-allelic expression (gain-of-function) of either *Igf2r* (*Igf2r*^+*m/R2Δ*^), due to paternal allele deletion of intron 2 *Airn* imprinting control region, or bi-allelic expression of *Igf2* (*H19*^*Δm*/+*p*^), due to disruption of CTCF binding region of the maternal allele *H19* imprinting control region, or both, significant respective alterations in body weight occurred as expected (Fig. 6a). Compared to wild-type littermates, bi-allelic expression of *Igf2* and *Igf2r* resulted in increased (p = 0.002) and decreased body weight (p = 0.0326), respectively. When *H19*^*Δm*/+*p*^ and *Igf2r*^+*m/R2Δ*^ were then combined, body weight normalised and was not significantly different from that of wild-type controls (p = 0.2174, Fig. 6a). The expectation based on our previous work in *Apc*^*Min*/+^, suggested that the number of intestinal adenoma (normalised to growth by using small intestinal surface area) would be similarly altered with the changes in size and body weight^20,37^. In *Apc*^*Min*/+^, *H19*^*Δm*/+*p*^, there appeared no significant increase in adenoma number, whereas in *Apc*^*Min*/+^, *Igf2r*^+*m/R2Δ*^ there was a significant reduction in the middle part of the small intestine (p = 0.0187), compatible with *Igf2r* having adenoma suppressor function. Paradoxically, when *Apc*^*Min*/+^, *H19*^*Δm*/+*p*^, *Igf2r*^+*m/R2Δ*^ were then combined, significant adenoma progression was observed, with a marked increase in both adenoma number and variation compared to wild-type littermates especially in the distal small intestine (p = 0.0183, Fig. 6b,c). Potential explanations for these unexpected results may be; segregating modifier loci of the *Apc*^*Min*/+^ adenoma phenotype resulting in variation, despite backcrossing mice to C57Bl6/J >10 generations, and alterations in miRNA that associate with genetic disruption of these loci. In order to circumvent some of these potential confounders, we next combined conditional *Apc*^+/*loxp*^ and *Igf2r*^*loxp/loxp*^ loss of function alleles with intestinal specific Cre transgenes.

### Combinations of Igf2r alleles with conditional Apc loxp supports Igf2r function as a tumour suppressor

Following constitutive expression of villin-Cre, the survival of mice (up to 400 days), and the number and growth of intestinal adenoma were unaltered with respect to either the presence or absence of *Igf2r*^*loxp/loxp*^ (see Supplementary Fig. S5a–c). Histological examination of small intestinal adenoma showed no phenotypic differences. A similar result occurred when *Igf2r*^*loxp/loxp*^ was combined with homozygote *Apc*^*loxp/loxp*^ using tamoxifen inducible Lgr5-Cre (*Lgr5CreER*^*T2*^) (see Supplementary Fig. S5d–f). Here, overall adenoma formation in adult mice was more rapid in onset (survival up to 40 days) because of the homozygous disruption (floxing) of the *Apc* alleles. Again, histological confirmation with anti-IGF2R antibodies, suggested that there was mosaic loss of *Igf2r* following conditional disruption in adenoma (see Supplementary Fig. S5g). This result suggests the potential for low floxing efficiency of the *Igf2r*^*loxp/loxp*^ alleles in intestinal adenoma, that may have masked our ability to quantify significant phenotypic differences.

**Figure 7:**
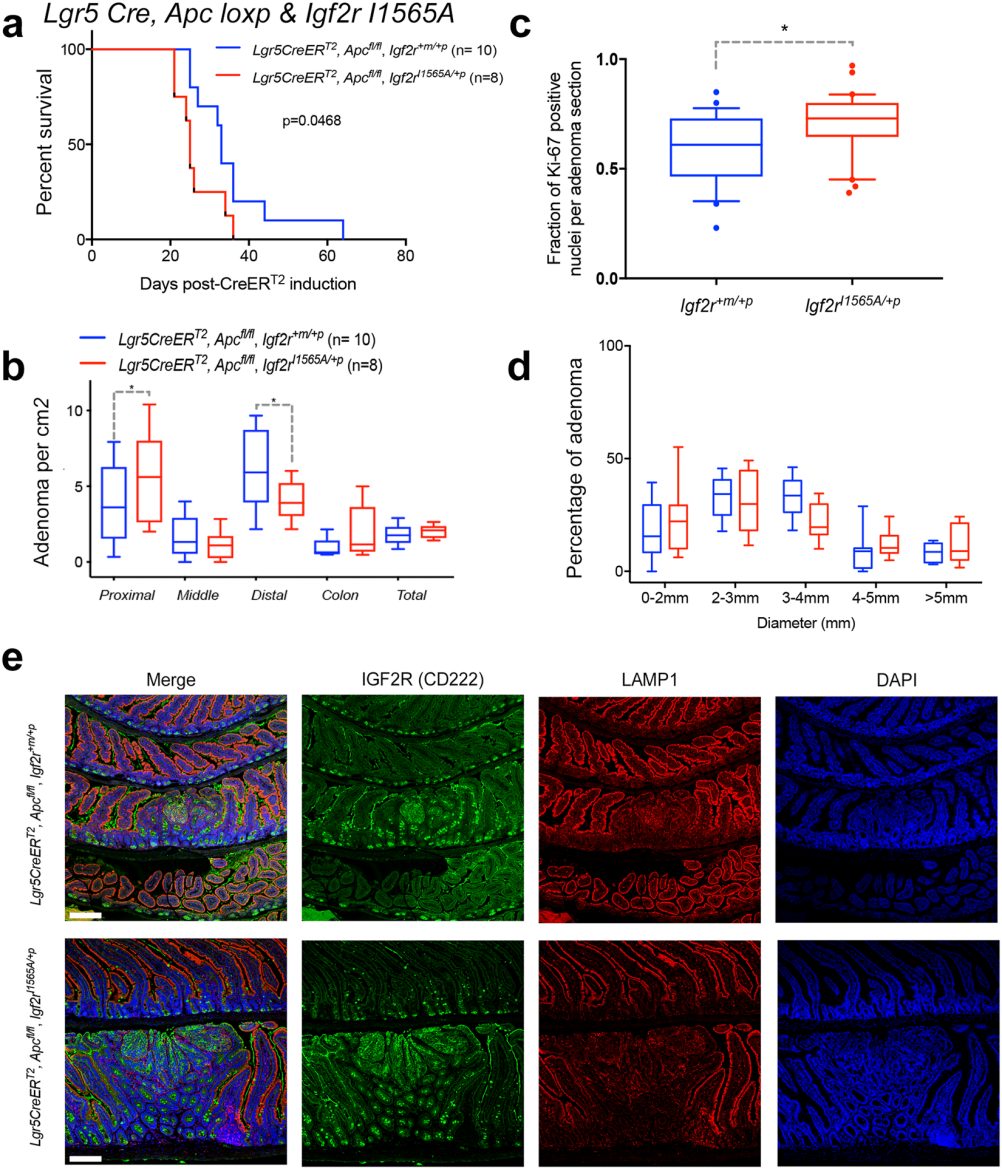
Loss of function of *Igf2r* (*Igf2r*^*I1565A*/+*p*^) shortens survival and promotes intestinal adenoma proliferation in conditional *Apc*^*loxp/loxp*^. The effects of *Igf2r*^*I1565A*/+*p*^ was determined following conditional generation of intestinal adenoma. *Igf2r*^*I1565A*/+*p*^ was first combined with homozygote *Apc*^*loxp/loxp*^, and an inducible intestinal Lgr5-Cre (*Lgr5CreER*^*T2*^). Tamoxifen injection induced Cre recombinase (injection at 6–8 weeks of age) in these mice, with survival and intestinal adenoma formation quantified. (**a**) Significant shortening of survival was observed (upto day 65) for *Igf2r*^*I1565A*/+*p*^ compared to *Igf2r*^+*m*/+*p*^ (Kaplan-Meier plot, long-rank test p = 0.0468). (**b**) Intestinal adenoma number in the distal small intestine was significantly lower in number when comparing *Igf2r*^*I1565A*/+*p*^ to *Igf2r*^+*m*/+*p*^ (*Distal p = 0.0149), whereas in the proximal small intestine they were signgicantly greater (*Proximal p = 0.0469). The differences in colonic adenoma between genotypes were not significant. All comparisons One-way ANOVA with Fishers LSD. (Note colonic adnoma differences were borderline with an unpaired one-tailed t-test, p = 0.0423). (**c**) Ki-67 proliferation labelling was significantly higher (p = 0.0302) in adenoma from *Igf2r*^*I1565A*/+*p*^ compared to *Igf2r*^+*m*/+*p*^ mice (Mann Whitney, two-tailed test). (**d**) Adenoma diameter were not significantly different between *Igf2r*^*I1565A*/+*p*^ compared to *Igf2r*^+*m*/+*p*^. (Two-way ANOVA). Percentage comparisons relative to total adenoma number per mouse. (**e**) No significant differences in the immuno-localisation of IGF2R protein were observed between *Igf2r*^*I1565A*/+*p*^ and *Igf2r*^+*m*/+*p*^ adenoma, in relation to nuclei (DAPI) and LAMP1 (endosomal compartment). Scale bar 100 μm.

We next combined homozygote loss of *Apc* (*Lgr5CreER*^*T2*^, *Apc*^*loxp/lox*^) on a *Igf2r*^*I1565A*/+*p*^ background, and injected tamoxifen into the surviving adult mice. By making the *Igf2r* loss of function IGF2 specific and constitutive within all intestinal cells with our new knock-in model, we then observed phenotypic differences that were *Igf2r*^*I1565A*/+*p*^ dependent. Significantly, we observed a decreased survival (log-rank, 0.0468), a shift to more proximal small intestinal adenoma (p = 0.0469) and an increase in distal small intestinal adenoma (p = 0.0149), with increased Ki-67 labelling of small intestinal adenoma (p = 0.0302), when comparing to *Lgr5CreER*^*T2*^, *Apc*^*loxp/loxp*^ littermate controls (Fig. 7a-d). The introduction of *Igf2r*^*I1565A*/+*p*^ did not alter the distribution of IGF2R protein within adenoma as expected (Fig. 7e). These data suggest that the constitutively expressed domain 11 specific knock-in mutation *Igf2r*^*I1565A*/+*p*^, supports the function of *Igf2r* as a tumour suppressor in the *Apc* dependent intestinal adenoma conditional model.

## Discussion

Phenotypic models of IGF pathway related genes have tended to exhibit gene dosage effects in keeping with some genes being imprinted, such as *Igf2* and *Igf2r*. In addition, haplo-insufficiency and hypomorphic alleles of the signalling receptor *Igf1r* have effects in relation to aging^38–41^. Aside from allelic dosage, the physiological genetic variants that modify *Igf2r* function remain poorly understood. Although synonymous and non-synonymous polymorphisms of *IGF2R* are known in human, there remains some debate as to whether they have direct functional effects on IGF2R^42–49^. Thus, to date there have been no specific point mutations introduced into the mouse germ-line that result in specific loss of function of *Igf2r* protein domains.

The loss of function of *Igf2r* maternal expressed allele in mouse development suggest that both loss of mannose 6-phosphate binding and lysosomal enzyme supply may contribute to the disproportionate growth phenotype, as the mechanism of growth regulation of *Igf2* appears proportionate to the mechanisms of cell proliferation and cell death at embryonic day 9^32^. By disrupting splicing regulation of both the targeted allele and the endogenous mouse *Igf2r* allele, we previously generated a reduction in overall IGF2R protein levels and an associated loss of *Igf2r* function phenotype^6^. In this previous model, perinatal lethality was dependent on *Igf2*, and we showed that we could rescue the phenotype by expression of a wild-type endogenous *Igf2r* allele. These studies provided the impetus to develop more a targeted and specific consequence of a knock-in alanine mutation in a key single residue that disrupts the CD loop binding to IGF2 within the domain 11 IGF2-binding site of IGF2R in the mouse. As we have shown, this mutation results in loss of IGF2 binding capacity by at least 100-fold, and to IGF2 alone. The inheritance of the loss of function allele in the mouse only results in a phenotype when transmitted through the maternal germ-line, indicating the allele did not alter imprinting control. Moreover, the phenotype we describe also differs significantly from that of the previously reported with complete loss-of-function *Igf2r* alleles, as a significant proportion of neonates survived into adulthood with milder growth phenotypes. These data suggest that the contribution of other IGF2 independent functions of *Igf2r* contributed to the more severe phenotype associated with complete *Igf2r* allele disruption previously reported^4–10^. The additional functions of IGF2R implicated in the latter models may include the disruption of lysosomal protease clearance and trafficking, potential modification of TGFβ signalling and cleavage of IGFBPs by enzymes such as the PAPP-A protease, resulting in further bioavailable IGF2 that could also promote growth^14^. We have summarised the growth outcomes of our knock-in *Igf2r*^*I1565A*/+*p*^ allele in relation to the previous literature concerning genetic manipulation of *Igf2* and *Igf2r* on embryonic and placental growth phenotype in the mouse^50^ (Supplementary Fig. S6).

The assumption is that the increased autocrine, paracrine and endocrine supply of IGF2 contribute to the phenotype observed. In terms of actual quantification of free IGF2 within mouse embryonic tissue, our experience reflects the similar limited data reported in previous publications, that are often complicated by acid-athanol extraction methods that aim to remove IGFBPs prior to ELISA, RIA or Western blots. For example, previous IGF2 quantification of IGF2 are selectively presented in either Western blots^51^, display limited sample numbers without presenting variation in raw data^5,8^, or only detected differences at selected embryonic time points (e.g. E12.5), with circulating IGF2 determined by radio-immunoassay^7^. The use of immuno-histochemistry to detect IGF2 within cells may be informative, but when the underlying mechanism is through extra-cellular ligand bio-availability, quantification of the soluble pool of IGF2 would be problematic using this method in washed tissue sections. These reports highlight the lack of sensitive analytical methods (such as *in situ* pulse chase methodology) to quantify the real time metabolism of IGF2 intra- and extra-cellularly, both in terms of its expression and delivery, but also its signaling receptor kinetics and receptor-ligand internalisation.

Our phenotypic data confirm that *Igf2r* domain 11 has an essential developmental role in IGF2 regulation, and that a single point mutation in a wild-type allele of *Igf2r* renders the protein unable to sequester IGF2, leading to over-growth of the embryo, placenta and heart, and subsequent early lethality. As a result, it is likely that hypomorphic *Igf2r* alleles with specific disruption of IGF2 binding function would not be expected to be evolutionary selected if they result in reduced viability of the offspring. It is tempting to speculate that one explanation for the subsequent loss of imprinting of *IGF2R* in human may reflect less selective evolutionary pressure to maintain mono-allelic expression, as the high IGF2 affinity achieved would indicate limited additional benefit of affinity enhancing mutations^2^.

Circumstantial data from sequencing human cancers and genetic modification of cell lines, have implicated IGF2R as a tumour suppressor gene^52–65^. Functionally defining the tumour suppressor function of IGF2R in mouse models *in vivo* has been dependent on the nature of the *Igf2r* gain and loss of function alleles, and the types of models used. For example, a constitutive YAC transgenic *Igf2r* gain of function model was shown to rescue *T*^*hp*^ (*Igf2r* loss of function), and also mammary tumour progression in an *Igf2* induced mammary tumour model^65^. This outcome mirrored that of over-expression of a soluble M6P/IGF2R transgene in the regression of intestinal adenoma in the *Apc*^*Min*^ model^20^. For loss of function, however, only a conditional disruption of *Igf2r* loxp alleles in the liver using an albumin-Cre has been reported, but failed to result in a (pre-) malignant phenotype^10^. These data also suggest that *Igf2r*, as with *Igf2*, regulates a second tumour signal in co-operation with a tumour initiating mutation^66^. Here we now show, using a Wnt pathway driven model, that existing models of *Igf2r* imprinting control disruption and the efficiency of conditional *Igf2r* allele floxing, limited our ability to generate informative loss and gain of-function models. By exploiting the partial survival of maternal allele transmission of *Igf2*^*I1565A*/+*p*^, we now show in a conditional early intestinal adenoma model (*Lgr5-Cre*, *Apc*^*loxp*^), that early adenoma growth can be modified by the IGF2 dependent function of *Igf2r*. These data suggest for the first time that early adenoma growth may be modified by the IGF2 dependent function of *Igf2r*, extending the potential that selection for IGF2 supply may be a functional driver in the development of colorectal cancer. Cancer genome (CGA) data confirms this hypothesis, with at least 20% of all colorectal cancers having associated increased IGF2 expression^67^. Whilst these data support the constitutive tumour suppressor function of *Igf2r*, it remains unknown what the specific cell type(s) are that express *Igf2r* in order to regulate tumour suppressor activity.

## Materials and Methods

### Mammalian protein expression and western blotting

Human Embryonic Kidney 293 (HEK293T) cells were grown in Dulbecco’s Modified Eagle Medium (DMEM; Sigma-Aldrich St Louis, MO, USA) supplemented with 10% fetal calf serum (FCS; Sigma-Aldrich St Louis, MO, USA) and 1% of penicillin & streptomycin (P&S; Life Technologies Ltd, Paisley, UK) at 37 °C in 5% CO_2_ incubator. Cells were passaged at 80–100% confluency using Gibco^®^ TrypLE™ Express (Life Technologies Ltd, Paisley, UK).

The 1–15 extracellular domains of human IGF2R were expressed in HEK293T cells as CD4 (domains 3 and 4) fusion protein cloned in the pHLsecAvitag with a 6His and BirA acceptor tags, as previously described^68^. A synthetic gene was created with the sequence altered to generate the I1572A mutant for human IGF2R (equivalent to mouse I565A), and cloned into the same expression vector following type IIS restriction enzyme digestion. HEK293T cells were also co-transfected with a plasmid for biotin ligase BirA expression and the medium was supplemented with 2 mM biotin. For transfection, DMEM medium (1.6 ml) was mixed with 36 µg of co-transfection expression vector mixture (34 µg of receptor expressing vector, 2 µg of biotin ligase expressing vector) followed by the addition of 144 μl of polyethylenimine (PEI) (1 mg/ml) followed by vortexing, incubation and addition to culture cells.

Biotinylated protein in the supernatant was purified using a PrepEase His-tagged protein purification kit (USB Affimetrix, UK). Proteins that were separated by PAGE were transferred to polyvinylidene fluoride (PVDF) membrane by wet transfer using MiniProtean 3 system, either at 100 V, at room temperature for an hour or at 30 V at 4 °C overnight, in transfer buffer composed of 14.4 g/L glycine, 3.02 g/L Tris and 10% methanol in double distilled water. The PVDF membranes were blocked and probed at room temperature for one hour or at 4 °C overnight. The membranes were washed with Tris-buffered saline with Tween 20 three times (TBS-T was 8.006 g/L NaCl, 2.423 g/L Tris base, 1% Tween20, pH 7.6). Blots were developed using ECL Plus Western Blotting detection system and Amersham Hyperfilm ECL (24 × 30 cm) from GE Healthcare Life Sciences (Little Chalfont, UK). Blocking solutions for PVDF membranes were prepared by dissolving either 3% bovine serum albumin (BSA Sigma-Aldrich St Louis, MO, USA) or 5% skimmed milk in Tris-Buffered saline with Tween 20 (TBS-T). Mouse Anti-His6 peroxidase conjugated monoclonal antibody from Roche (West Sussex, UK) was diluted 1 in 10000 in the blocking reagent to probe poly-histidine tag. To detect biotinylation, high sensitivity streptavidin HRP Conjugate from Pierce (Rockford, Il, USA) was diluted 1 in 20000 in the blocking reagent. Antibodies used included anti-IGF2R (R&D systems, AF2447).

### Surface plasmon resonance

Surface plasmon resonance was performed on a BIAcore® T200 for human IGF2 binding to human CD4 domains 3 and 4-IGF2R fusion proteins. For human IGF2 (7.4 KD) and IGF1 (7.7 KD) binding, approximately 1800 response units of biotinylated CD4-IGF2R fusion protein were immobilized on a CM5 chip previously loaded with Streptavidin by amine coupling. Approximately, 200 RU of the receptor was immobilised for higher molecular weight analytes such as bovine β-glucuronidase (290 KD) and human glu-plasminogen (88 KD). In presence of acetate ions, plasminogen exists in an open conformation, and so binding was performed in 150 mM sodium acetate. Kinetic binding experiments were carried out at 25 °C at a 40 μl/min flow rate in HBS-EP binding buffer by injecting solutions of IGF2 from both species ranging from 256 to 0.125 nM for 200 seconds. In order to avoid mass transport effects, dissociation constants (K_D_) were also confirmed at a range of flow rates (5, 10, 20, 40 and 80 µl/min), and indicated low K_D_ values at flow rates below 40 μl/min. Analyte solutions were then replaced by HBS-EP buffer for 1 hr and followed by a 60 μl injection of 2 M MgCl_2_ for regeneration of the sensor chip surface. A buffer control and a reference flow cell were included. Data were analysed using the BIAcore T200 Evaluation software version 1.0 and both by fitting the kinetic data to 1:1 binding model and by steady state analysis at equilibrium.

### Generation of the Igf2r^I1565A^ allele

All animal work was approved by University of Oxford animal ethics committee, and was performed under a UK Home Office Project Licence (ABH). All regulated procedures were performed according to guidelines, by trained staff. All severity levels and outcomes were monitored throughout, including humane endpoints for breeding and intestinal tumour monitoring. Genetic targeting of *Igf2r* to insert a point mutation (C57Bl/6NTac-*Igf2r*^*tm2760(I1565A)Arte*^) was generated in collaboration with Artemis-Taconic (Cologne, Germany, Fig. 2). Viable mice were generated bearing the knock-in allele on the paternal allele *Igf2r*^+*m/I1565A*^. Briefly, the pIgf2r-targeting vector was constructed using a PCR based cloning strategy using BAC clones from the C57BL/6J RPCI-731 BAC library (Fig. 2a). The flanking short arm (6.3Kb) contained exons 28–31, followed by a positive PuroR (Puromycin resistance) selection marker flanked by Frt sites into intron 31, the mutation in exon 34 (ATC to GCC to generate I1565A) and the long arm (10.3 Kb) that contained exons 35–42 followed by the thymidine kinase (Tk) negative selection marker. Artemis Taconic C57Bl/6N Tac ES cell lines were transfected and selected homologous recombination clones micro-injected into BALB/c blastocysts (Fig. 2b). The C57BL/6N Tac ES cell line growth and transfection were as described previously^6^. The final constitutive expressing allele was generated following breeding with Flp deleter mice (*Igf2r*^+*m/I1565A*^), to remove the PuroR, but retaining a single Frt site, used then for genotyping using flanking PCR primers to generate a larger product (302 bp) compared to the wild-type allele (227 bp) (Fig. 2c). Primers used were O3: GGTGCAGGTGTTATGACTGG and O4:AGTACATCAGCCTGTATCCTGG flanking the Frt site. Derived lines were bred against C57Bl/6 N.

### Mouse breeding

Two female and two male *Igf2r*^+*m/I1565A*^ mice from lines (A-G02, B-D03) were paired with C57BL/6J wild-type mice and the resulting offspring genotyped. *Igf2r*^+*m/I1565A*^ transmitted through the maternal germ-line resulted in identical frequency of neonatal lethality in resulting offspring (see also Results). Males from *Igf2r*^+*m/I1565A*^ A-G02 were paired with wild-type C57BL/6J female mice for all subsequent breeding. All other lines were obtained with permission and were backcrossed >10 generations onto C57BL/6J^9,69^. These lines were: *Igf2r*^*R2Δm*/+*p*^ line, bi-allelic expression of *Igf2r* resulting from disruption of intron 2 imprinting control region, the *H19*^*Δ13kb*/+*p*^ line resulting in bi-allelic *Igf2* expression following disruption of the CTCF boundary, the *Apc*^*Min*^ ‘line1’ spontaneous adenoma model^70^, *Igf2*^*lox/loxp*^ (*Igf2*^*tmConstancia*^), *Apc*^*loxp/loxp*^ (*Apc*^*tm1Tno*^) conditional loxp intestinal adenoma model^71^, *Vil-CreER*^*T2*^ (Tg(Vil-cre/ERT2)23Syr)^72^ and *Lgr5*^*tm1(cre/ERT2)Cle*^ (129P2/OlaHsd)^73^ tamoxifen inducible intestinal Cre lines and the cardiac *Nkx2.5Cre* (C57BL/6J) line. Embryos were staged by taking mid-day on the day of seminal plug detection as embryonic day 0.5 (E0.5). Weighed embryos were fixed in 4% neutral buffered formalin.

### PCR genotyping

Genotyping was performed by PCR using ear clips and standard conditions, as above and previously described^37^.

### Intestinal adenoma phenotype

Induction of Cre with tamoxifen and phenotyping of intestinal adenoma followed post-mortem dissection were performed as previously described^37^.

### IGF2 western blot quantification

In order to quantify levels of bio-available IGF2 in conceptus tissues exhibiting overgrowth phenotype, we dissected placental and embryos from litters collected at E16.5. Placenta, hearts and embryos were dissected apart in ice-cold PBS (4 °C), before weighing, freezing and storage at −80 °C. Whole embryos, hearts and placenta were subject to homogenisation as described^6^. Following SDS-PAGE without prior acid-ethanol extraction, the proteins and recombinant mouse and human IGF2 positive controls (Gropep) were then electro-transferred overnight at 4 °C to a PVDF membrane in a variety of buffers of different pH and 10–15% methanol, and the blots incubated with Ponceau S to verify equal protein loading. For detection, anti-IGF2 (R&D systems, AF792) at 0.4 µg ml^*−*1^ was used as the primary antiobody with an HRP-conjugated secondary antibody (Dako, 1:2000).

### Immunofluorescence

Tissue samples were fixed in 4% (v/v) neutral buffered formalin at RT for 24 hr, before dehydration and paraffin embedding. Sections (5 µm) were processed by standard techniques. Briefly, slides were de-waxed in xylene, rehydrated followed by antigen de-masking in sodium citrate buffer (10 mM, pH6.0) in a pressure cooker for 2 min at 125 °C and 10 min at 85 °C. Washed tissue sections (Tris-buffered saline, TBS, pH7.4) were blocked in 10% goat serum/Tween 20^©^ 0.5% (v/v) for 1 hr at RT followed by incubation with primary antibodies; anti-E-cadherin (mouse, 1:200, BD610182, BD laboratories, UK); anti-Ki-67 (rabbit, 1:300, RM9106, Thermo Scientific, USA), Anti-phospho histone H3 (pH3, mouse, 1:1000, Abcam), anti-CTip2 (rat, 1:400, Abcam,), anti-IGF2R (goat, 1:200, AF2447, R&D Systems) and anti-LAMP1 (rat, 1:100, sc19992, Santa Cruz) at 4 °C overnight. After 3 washes in TBS tissue sections were incubated with secondary fluorescent-conjugated antibodies (goat anti-mouse or goat anti-rabbit, Alexa-488, -555, -594, or -647, 1:300, Invitrogen, USA, or goat anti-chicken dylight-550, Abcam, UK) for 2 hr at RT or biotinylated antibodies as per manufacturer’s instructions (Vectastain elite avidin-biotin complex kit, Vector Laboratories). After washing, fluorescent-labelled tissue sections were counterstained with DAPI (1:5000, D9663, Invitrogen, USA) and mounted with Prolong gold anti-fade reagent (P36930, Invitrogen, USA). Fluorescent images were acquired using a confocal microscope Olympus Fluoview FV1000. Sections labelled using a biotinylated secondary antibody were counterstained with haematoxylin, dehydrated through an alcohol series and mounted with Depex mounting medium (Electron Microscopy Sciences, USA).

### Statistical analysis

All statistical analysis utilised Prism Version 8 software. For comparison of conceptus and organ growth by genotype, a one-way ANOVA with pairwise comparisons with wild-type controls were performed where stated with either Bonferroni multiple comparisons test for adjusted p-values, or where stated, with Fishers LSD. For Kaplan-Meier survival analysis, the log-rank test (Mantel-Cox), and for non-parametric comparison, the Mann-Whitney test, were applied, respectively.

## Supplementary information

Supplementary Information Hughes et al

## Data Availability

All primary data reported in this manuscript is accessible by contacting the corresponding author.
