# Supplementary material for: Maternal transmission of an Igf2r domain 11: IGF2 binding mutant allele (Igf2rI1565A) results in partial lethality, overgrowth and intestinal adenoma progression
Source: Sci Rep. 2019 Aug 6;9:11388. doi: 10.1038/s41598-019-47827-9 (PMC6684648; doi:10.1038/s41598-019-47827-9)
Supplement: Supplementary file 1 — Supplementary Information Hughes et al [file 41598_2019_47827_MOESM1_ESM.pdf]

Supplementary Information

Maternal transmission of an *Igf2r* domain 11: IGF2 binding mutant (*Igf2r*$^{\mathbf{I1565A}}$) results in partial lethality, overgrowth and intestinal adenoma progression.

Jennifer Hughes[1]*, Mirvat Surakhy[1]*, Sermet Can[1*], Martin Ducker[2], Nick Davies[2], Francis Szele[2], Claudia Buehnemann[1], Emma Carter[1], Roman Trikin[1], Matthew P. Crump[3], Susana Frago[1] and A. Bassim Hassan[1¶]

Supplementary Table S1

Summary of homozygote breeding (*Igf2r*$^{+m/I565A}$ ♀ x *Igf2r*$^{+m/I565A}$ ♂) outcomes in utero

Stage	Genotypes	Observed	Expected	
	Igf2r$^{+m/+p}$	4	2.25	
E14.5	*Igf2r*$^{+m/I565A}$ *or* *Igf2r*$^{I565A/+p}$	3	4.5	P=NS
	Igf2r$^{I565A/I565A}$	2	2.25	
	Igf2r$^{+m/+p}$	4	5.75	
E17.5	*Igf2r*$^{+m/I565A}$ *or* *Igf2r*$^{I565A/+p}$	7	9.5	P=NS
	Igf2r$^{I565A/I565A}$	8	4.75	
	Igf2r$^{+m/+p}$	4	3.75	
E18.5	*Igf2r*$^{+m/I565A}$ *or* *Igf2r*$^{I565A/+p}$	6	7.5	P=NS
	Igf2r$^{I565A/I565A}$	5	3.75	
	Igf2r$^{+m/+p}$	12	10.75	
All Embryos	*Igf2r*$^{+m/I565A}$ *or* *Igf2r*$^{I565A/+p}$	16	21.5	P=NS
	Igf2r$^{I565A/I565A}$	15	10.75	

X^2 and one-way ANOVA, Kruskal-Wallis, with Dunns multiple comparison post-test.

Supplementary Figure S1

Southern Blot Analysis

Homologous recombination at the 5' side

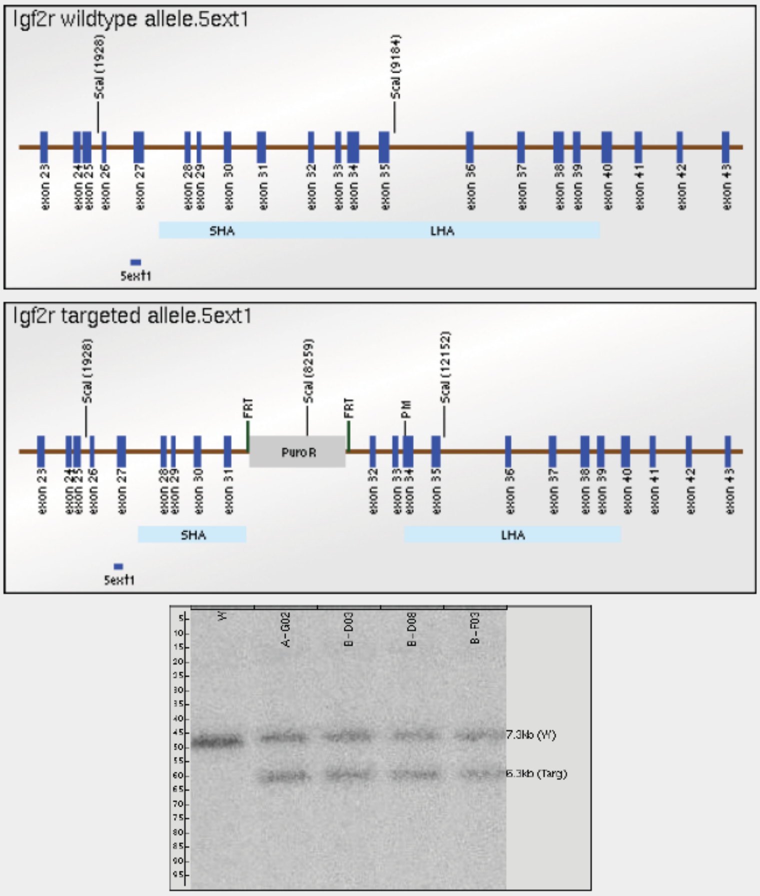

Material: Genomic DNA from W, A-G02, B-D03, B-D08, B-F03.

Method: Digestion with: ScaI, Probe: 5ext1

Results: Detects correct homologous recombination at the 5' side in all clones.

Locus and southern blot of 5'probe 5ext1 shown in Figure 2b.

Supplementary Figure S2

Homologous recombination at the 3' side

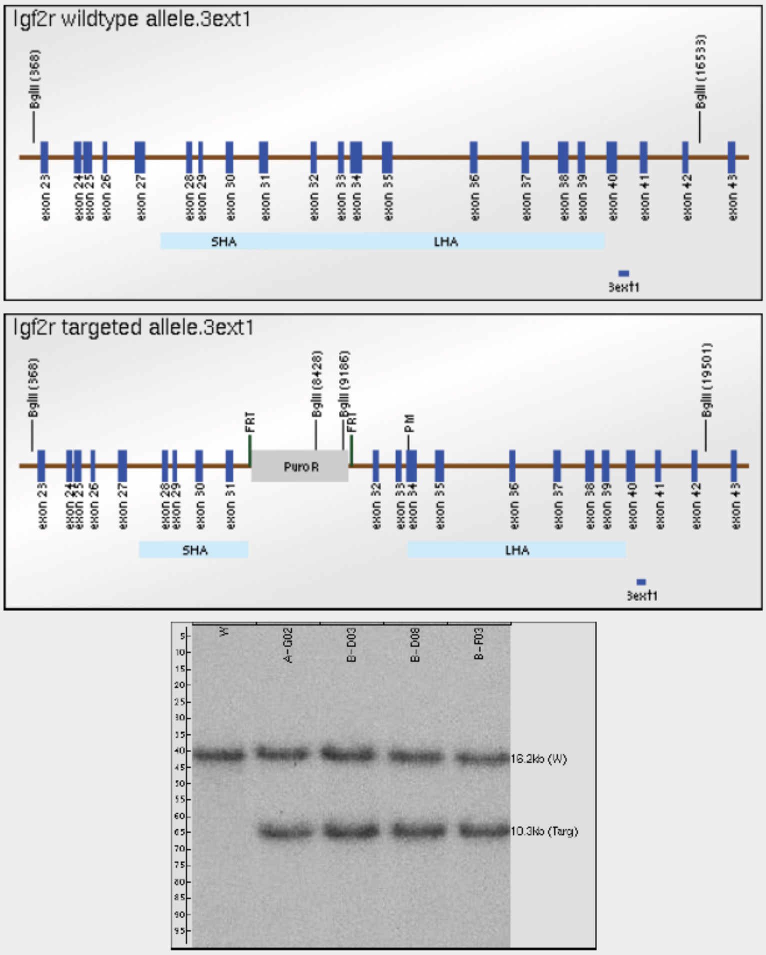

Material: Genomic DNA from W, A-G02, B-D03, B-D08, B-F03.

Method: Digestion with: BglII, Probe: 3ext1

Results: Detects correct homologous recombination at the 3' side in all clones.

Locus and southern blot of 3'probe 3ext1 in Figure 2b.

Supplementary Figure S3

Homologous recombination at the 5' side and single integration

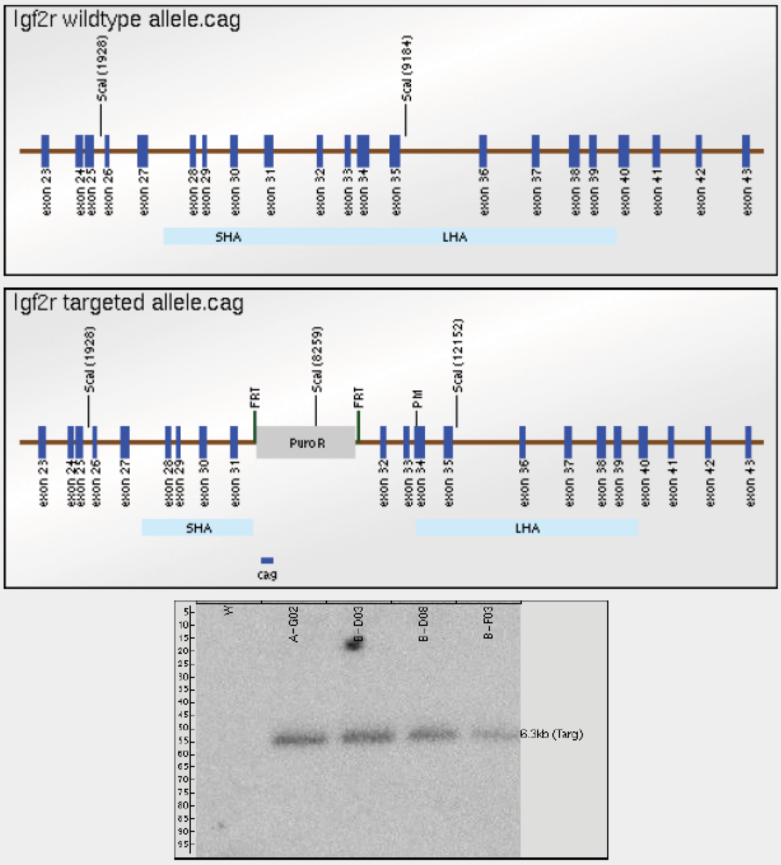

Material: Genomic DNA from W, A-G02, B-D03, B-D08, B-F03.

Method: Digestion with: ScaI, Probe: cag

Results: Detects correct homologous recombination at the 5' side and single integration in all clones.

Southern blot of probe cag in Figure 2b.

Supplementary Figure S4

PCR Analysis

PCR Analysis According to PCR SOP 5558

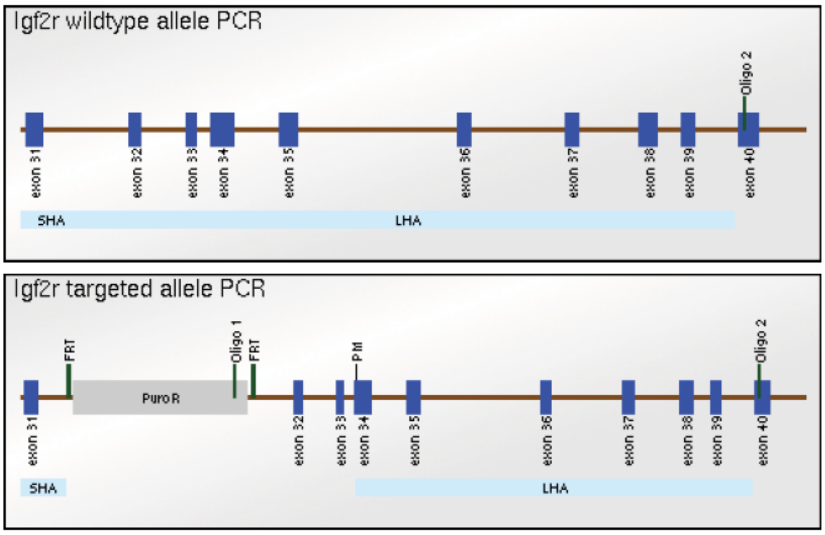

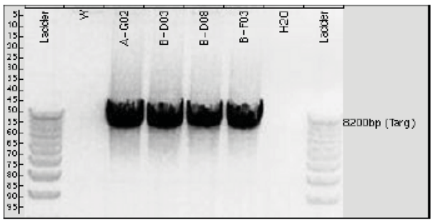

Igf2r PCR

The insertion of the point mutation (I1565A) was detected in all targeted clones by sequencing the PCR products.

oligo1=5558_1_Puro_F2
oligo2=5558_2_Igf2r_28

PCR genotyping for Figure 2c.

Supplementary Figure S5

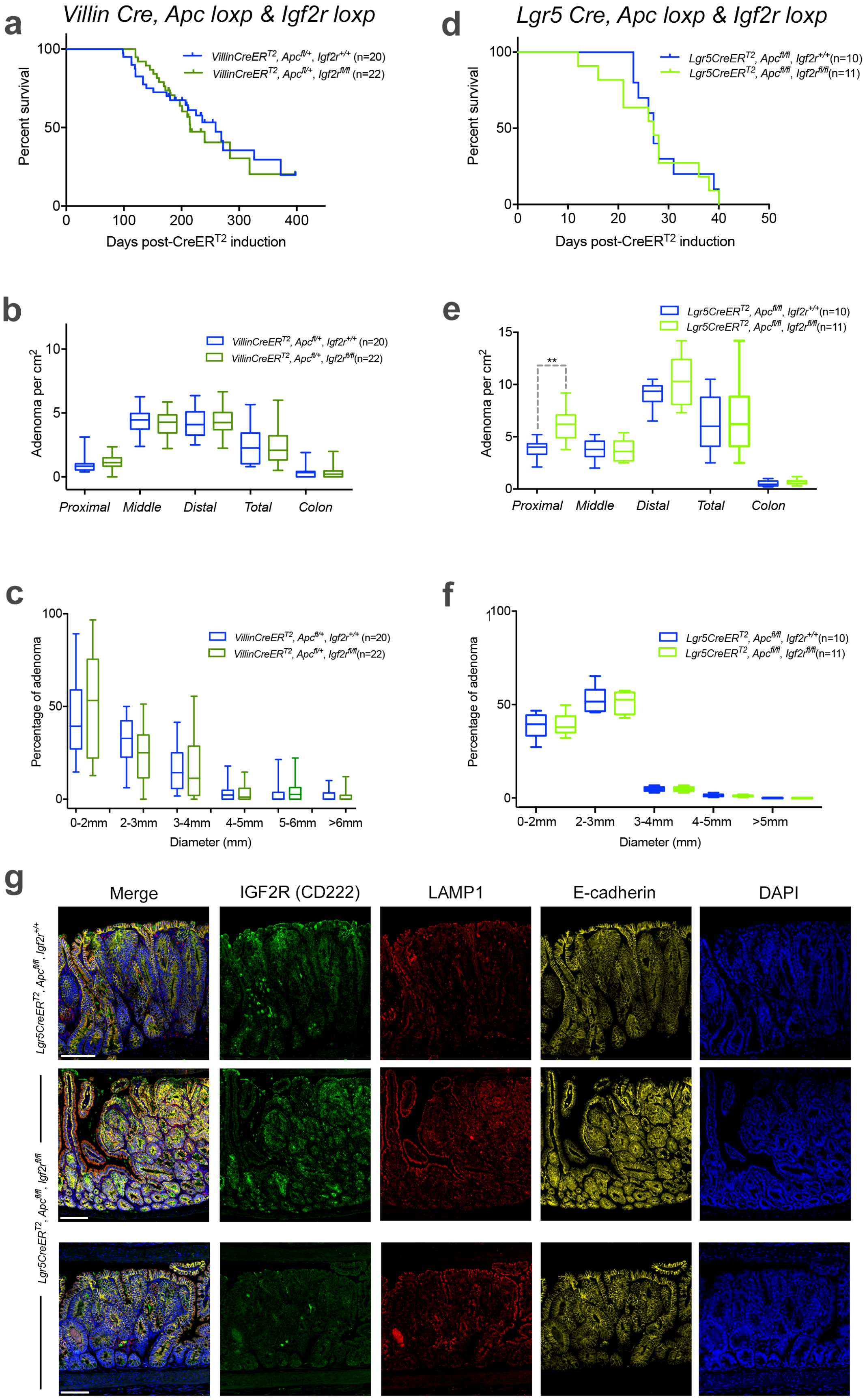

Loss of function of *Igf2r* (*Igf2r*$^{loxp/loxp}$) has limited impact on *Apc*$^{loxp/loxp}$ intestinal adenoma because of inefficient floxing.

(a, b, c) The effects of conditional loss of function of *Igf2r* (*Igf2r*$^{loxp/loxp}$) combined with heterozygote *Apc*$^{+/loxp}$ using tamoxifen (injection at 6-8 weeks of age) inducible villin-Cre (*Vil-CreER*^{T2}). Note the non-significant differences in survival (Kaplan-Meier) up to 400 days in **a**., intestinal adenoma number in the small intestine (proximal, middle and distal thirds) and colon in **b**., and adenoma diameter in **c**., between floxed *Apc* heterozygote without (control) and with homozygote floxed *Igf2r*. **(d, e, f)** The effects of conditional loss of function of *Igf2r* (*Igf2r*$^{loxp/loxp}$) combined with homozygote *Apc*$^{loxp/loxp}$ using tamoxifen (injection at 6-8 weeks of age) inducible Lgr5-Cre (*Lgr5CreER*^{T2}). Note the non-significant differences in survival up to 40 days (Kaplan-Meier) in **d**., intestinal adenoma number in the small intestine (proximal significant unlike, middle and distal thirds) and colon in **e**., and adenoma diameter in **f**., between floxed homozygote *Apc* without (control) and with homozygote floxed *Igf2r*. **g.** Immuno-localisation of IGF2R compared to nuclei (DAPI), LAMP1 (endosomal compartment), E-cadherin (adherence junctions) in adenoma from *Lgr5CreER*^{T2}, *Apc*$^{loxp/loxp}$, *Igf2r*$^{loxp/loxp}$ in **d.** and *Igf2r*$^{+/+}$control mice. Note the variable (mosaic) conditional loss of IGF2R labelling following adenoma formation. Bar 100μm.

Supplementary Figure S6

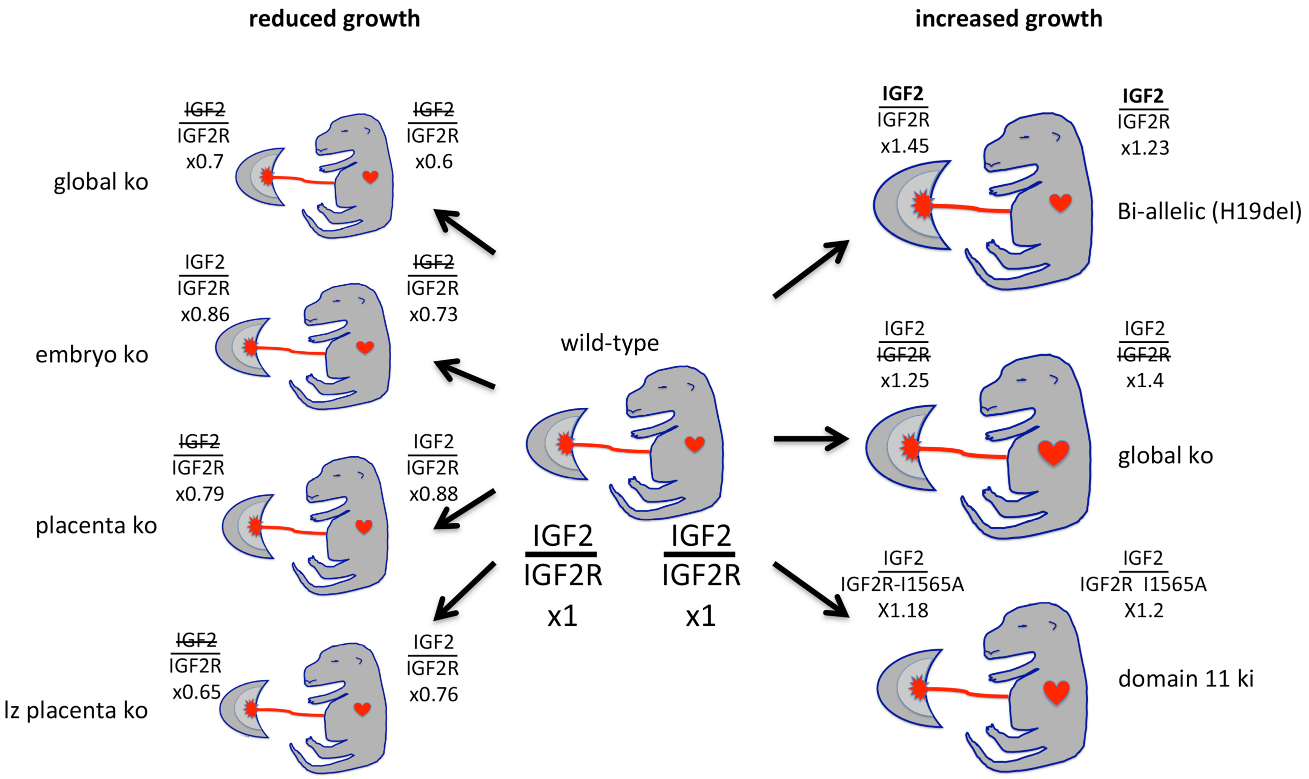

Summary: the effects of *Igf2* and *Igf2r* genetic manipulation on the growth of embryo and placental in the mouse at E18.5.
Representation of placental and embryo growth in the mouse expressed as the ratio of growth promotion (*Igf2*) over growth inhibition (*Igf2r*) genes relative to wild-type (=1). Values obtained from the literature as summarised by [1] including references therein, and reported for data at E18.5. Reduced growth secondary to less IGF2 can occur following a global, embryo specific, placental specific or labyrinth zone (lz) *Igf2* knockout (P0 promoter) of the *Igf2* gene, affecting both embryo and placenta, and resulting in disproportionate reduced growth. For overgrowth, bi-allelic expression of *Igf2* (bold) has proportionate effects on the placenta and embryo, yet global loss of function of *Igf2r* results in disproportionate overgrowth. $Igf2r^{+m/I565A}$ results in an attenuated over-growth phenotype compared to the global knockout. As the latter is a 'pure IGF2' supply effect, other functions of the receptor and/or consequences of the genetic disruption may have complicated all the other genetic models that also involve co-disruption of miRNAs and the genomic locus.

Sferruzzi-Perri, A. N., Sandovici, I., Constancia, M. & Fowden, A. L. Placental phenotype and the insulin-like growth factors: resource allocation to fetal growth. *J Physiol* **595**, 5057-5093, (2017).
